# Supplementary figures and images for: Elucidate microbial characteristics in a full-scale treatment plant for offshore oil produced wastewater
Source: PLoS One. 2021 Aug 12;16(8):e0255836. doi: 10.1371/journal.pone.0255836 (PMC8360554; doi:10.1371/journal.pone.0255836)

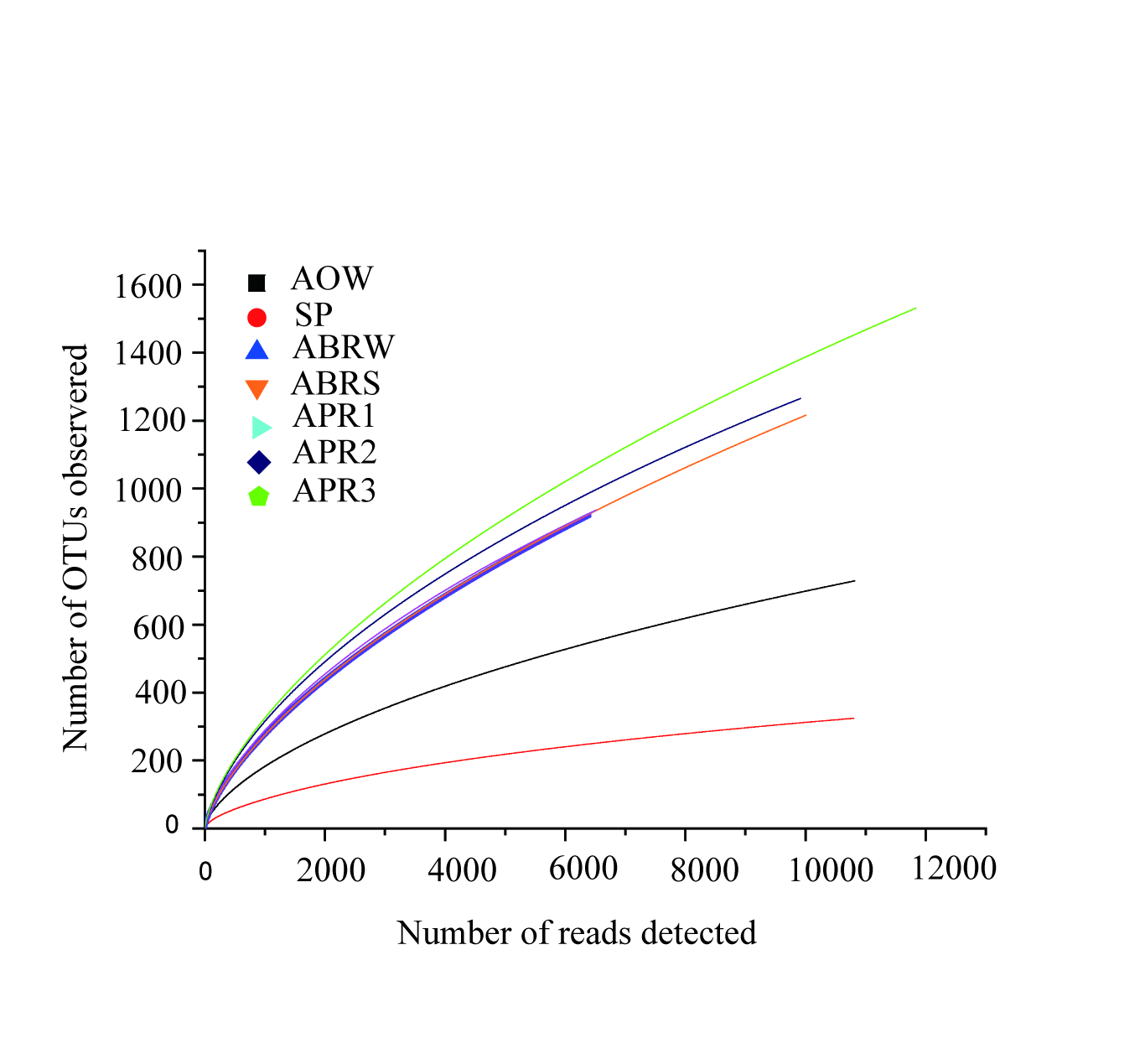

Supplement: S1 Fig — The bacterial phylogenetic tree of the dominant OTUs was constructed using the neighbor-joining method. Scatter points showing the relative abundances (%) of reads for each dominant OUT detected in the three samples. Samples were shown in different colors. (TIF) [file pone.0255836.s001.tif]

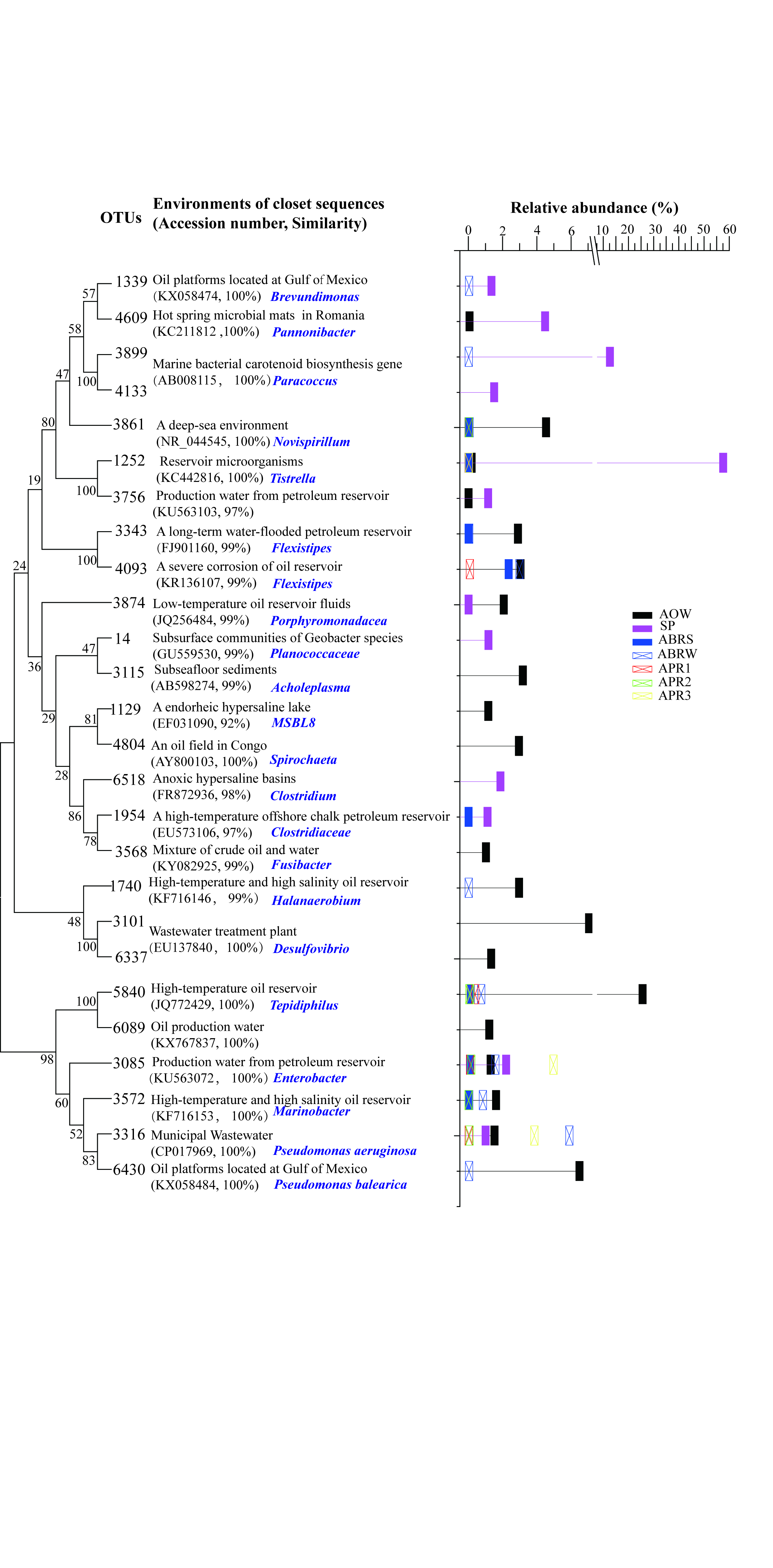

Supplement: S2 Fig — The bacterial phylogenetic tree of the dominant OTUs was constructed using the neighbor-joining method. Scatter points showing the relative abundances (%) for reads of each dominant OUT detected in the three samples. Samples were show in different colors. (TIF) [file pone.0255836.s002.tif]

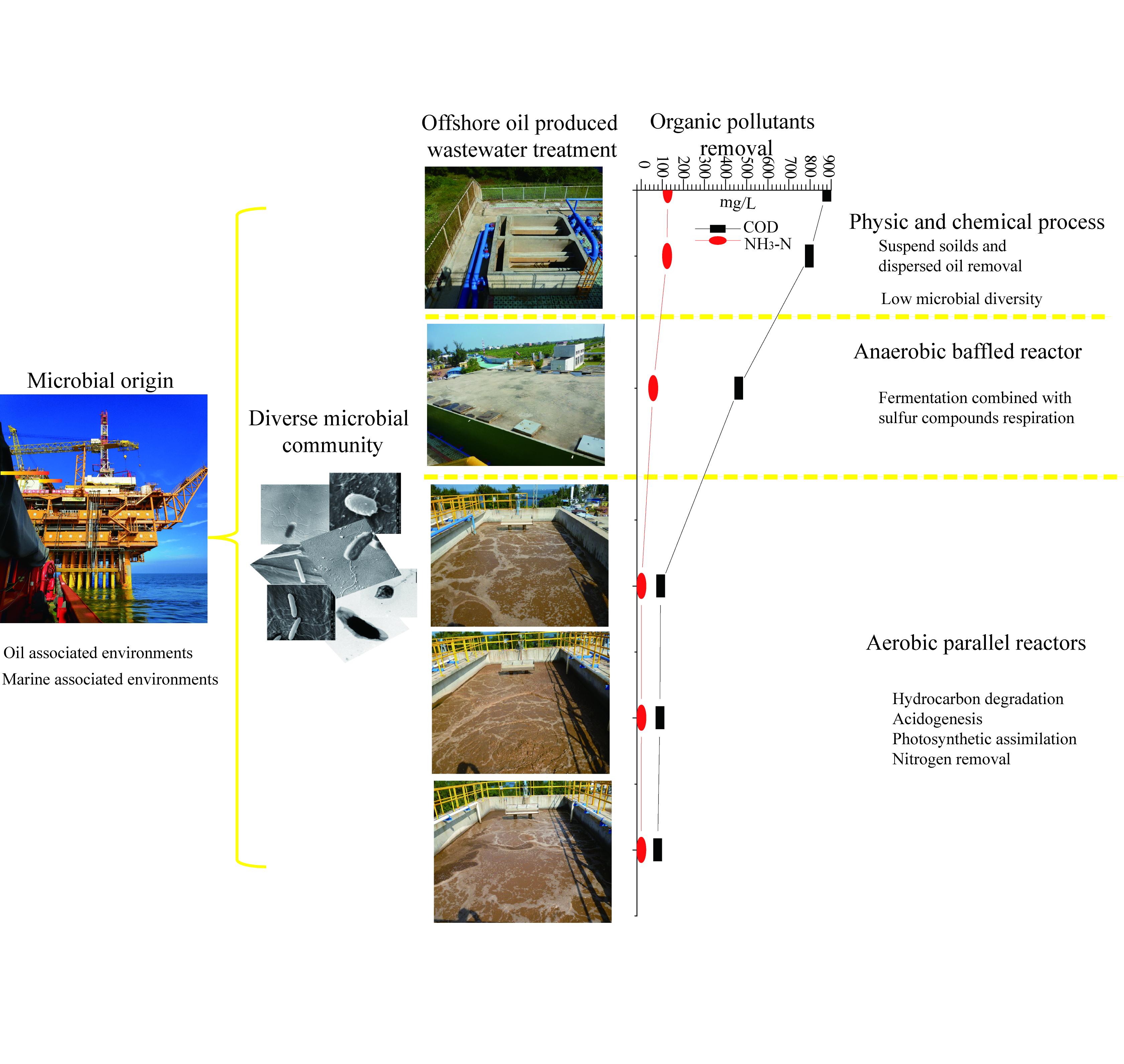

Supplement: S1 Graphical abstract — (TIF) [file pone.0255836.s003.tif]
