# Supplementary material for: Elucidate microbial characteristics in a full-scale treatment plant for offshore oil produced wastewater
Source: PLoS One. 2021 Aug 12;16(8):e0255836. doi: 10.1371/journal.pone.0255836 (PMC8360554; doi:10.1371/journal.pone.0255836)
Supplement: Attachment — Submitted filename: Comments.docx [file pone.0255836.s004.docx]

Ref: PONE-D-21-08773

Title: Elucidate microbial characteristics in a full-scale treatment plant for offshore oil produced wastewater

This manuscript elucidated the microbial composition and the potential microbial functions in a full-scale well-worked offshore oil produced wastewater treatment plant using 16S rDNA amplicon sequencing. In general, the finding of this work is interesting. However, the following comments should be addressed.

(1) The level of English should be improved and the data format should be unified throughout the manuscript.
(2) In “Highlight” sections, their content should be rewritten and simplified for better description the manuscript.
(3) If the abbreviation appears for the first time in the manuscript, please indicate its specific name. (like VFA, WWTS)

(4) “NH3-N concentration in the water phase of ashore water-oil sample was 125.58 mg/L, after the anaerobic treatment the NH3-N concentration decreased to 56.72 mg/L”. Please explain how NH3-N was removed in ABR.

(5) Concentration of sulfate was increased in ABR, why? How COD was removed in ABR? Why was not methane produced in ABR?

(6) What was the physical processes?

(7) The salt concentration in the oil production wastewater is high. In the process of biological treatment of wastewater, how much influence does it have on the treatment efficiency of microorganisms?

(8) In three aerobic reactors, the diversity and functional similarities of microorganisms in three different aerobic treatments need to be discussed in more detail.

(9) The layout of Figure 3 May be improve and some signs overlap so the signs are not clear.

(10) Please check “A majority of sequences detected by highthrough sequencing methods are identified with bacteria below to order lever that can not be affiliated with functional groups as many functions are only conserved at species or genus

level in FAPROTAX database. I”.
