# Supplementary material for: Elucidate microbial characteristics in a full-scale treatment plant for offshore oil produced wastewater
Source: PLoS One. 2021 Aug 12;16(8):e0255836. doi: 10.1371/journal.pone.0255836 (PMC8360554; doi:10.1371/journal.pone.0255836)
Supplement: Attachment — Submitted filename: Review_SR.docx [file pone.0255836.s005.docx]

Review for PONE-D-21-08773

In this study authors describe the biological treatment of oil-produced wastewater. To improve the treatment process and meet stringent effluent discharge limits, it is important to identify the microbial members found in these systems and understand their metabolic potential. The topic is interesting, the experiments are comprehensive, the findings are useful, for adding in-depth understanding to biological wastewater treatment. To make it better, I have some suggestions/questions as below:

Pg 3, ‘…remains curtail to modern civilization..”

- Sentence unclear

Pg 5, In this work, we elucidated the structures and potential functions of bacteria in a full-scale offshore oil produced wastewater treatment plant based on highthrough 16S rDNA sequences.

- Do the authors mean 16S ribosomal RNA (rRNA) or recombinant DNA (rDNA)?

Pg 6, correct OIIME-1.6.0 Pipelines to QIIME-1.6.0…

Pg 8, ‘NH3-N concentration in the water phase of ashore water-oil sample was 125.58 mg/L, after the anaerobic treatment the NH3-N concentration decreased to 56.72 mg/L, and after aerobic treatment the NH3-N concentration in the effluent below 1 mg/L (Table 1).’

- Ammonia usually has an inhibitory effect on anaerobic digestion but given that 334 mg/L COD reduction was observed, it might seem that the ammonia was either being stripped in gas phase or some microorganisms present in the digester could utilize ammonia since in section 3.4 there is mention of oxygen intrusion?

Pg 8, Section 3.2, It is important to statistically analyse changes in abundance of microbial community members (Fig 2) and metabolic functions (Fig 3) in different treatments and include it in discussions of sections 3.2 – 3.5.

Pg 9, ‘In addition, as seen in Fig. S1, only the rarefaction curve of AOW and SP tended to approach the saturation plateau. The rest of rarefaction curves show little sign of plateau, indicating that the biological treatment processes contained a somewhat broader phylogenetic diversity...’

- α-, β diversity measures will be more conclusive than rarefraction curves. Rarefraction curves not plateauing may also refer to insufficient depth of sequencing.

Pg 12, ‘…which could provide microbial data to the downstream treatment system, especially to those systems using biotreatments.’

- Sentence unclear

Pg 16, However, the aerobes and anaerobes co-existing ecology systems were reported popular in natural environments and could significantly enhance pollutants removal...

Pg 17, …as organic matters from wastewater are initially adsorbed to the activated sludge and are degraded by microbes inhabited in the inner of the sludge (Mario, et al., 2019).

- Above explanations can be improved by discussing factors that lead to ecological gradients in sludge and how this allows microorganisms to co-exist in these conditions.

Pg 20 - Hyphomicrobiaceae-affiliated microbes inhabited in wastewater system

were commonly detected to posse nitrogen removal enzymes and were closely related to

nitrogen removal (Jia et al., 2019; Tomasek et al., 2017).

- Authors may also discuss that Hyphomicrobium-related species are also commonly found in denitrifying wastewater treatment systems as identified in:

a) Lu, H., Chandran, K. and Stensel, D., 2014. Microbial ecology of denitrification in biological wastewater treatment. *Water research*, *64*, pp.237-254.

Pg 20 – “However, as the microbial communities of the studied reactors were so complex and consisted of a large percentage of uncultured candidate divisions, it was not easy to draw the potential metabolize network in each reactor. More studies associated strains isolation and function genes detection are therefore important to follow up.”

- Authors can mention that since many microorganisms could not be identified, it should be considered in future work on this topic. Further, strain isolation has several redundancies but new techniques such as long-read sequencing methods are being increasingly applied to identify microbial community members in wastewater treatment plants as shown in:

a) Singleton, C.M., Petriglieri, F., Kristensen, J.M., Kirkegaard, R.H., Michaelsen, T.Y., Andersen, M.H., Kondrotaite, Z., Karst, S.M., Dueholm, M.S., Nielsen, P.H. and Albertsen, M., 2021. Connecting structure to function with the recovery of over 1000 high-quality metagenome-assembled genomes from activated sludge using long-read sequencing. *Nature Communications*, *12*(1), pp.1-13.
b) Arumugam, K., Bessarab, I., Haryono, M.A.S. *et al.* 2021. Recovery of complete genomes and non-chromosomal replicons from activated sludge enrichment microbial communities with long read metagenome sequencing. *npj Biofilms Microbiomes* **7**, Nature Publishing Group, https://doi.org/10.1038/s41522-021-00196-6

Pg 20 – To increase the impact of this work and strengthen its relevance in the environmental context, the authors can briefly discuss the potential impact of climate change and rising temperature on the overall function of oil produced wastewater treatment plants since they are mostly inoculated with marine/oil sources. To assist the authors, there has already been some recent discussion regarding the impact of climate change on wastewater treatment and mitigation strategies, e.g.:

a) Lu, L., Guest, J.S., Peters, C.A., Zhu, X., Rau, G.H. and Ren, Z.J., 2018. Wastewater treatment for carbon capture and utilization. *Nature Sustainability*, *1*(12), pp.750-758.
b) Qu, J., Wang, H., Wang, K., Yu, G., Ke, B., Yu, H.Q., Ren, H., Zheng, X., Li, J., Li, W.W. and Gao, S., 2019. Municipal wastewater treatment in China: Development history and future perspectives. *Frontiers of Environmental Science & Engineering*, *13*(6), pp.1-7.
c) Qiu, G., Law, Y., Zuniga-Montanez, R., Lu, Y., Roy, S., Thi, S.S., Hoon, H.Y., Nguyen, T.Q.N., Eganathan, K., Liu, X. and Nielsen, P.H., 2021. Global warming readiness: Feasibility of enhanced biological phosphorus removal from wastewater at 35oC. *bioRxiv*.

Pg 20 – Conclusion: In this study, we have characterized the composition and interaction of the bacteria inhabiting in the full-scale…

>In my understanding, no interactions have been mentioned in the manuscript, rather the functional potential is discussed.

Table 1. Correct to AWO

Fig 1. Can mention in footnote that 2 samples, ABRS and ABRW, were collected from ABR.

Fig 2. Different colours to be used for alphaproteobacteria and unidentified taxa.

References – Please check the consistency of all references, including author names, journal names and titles.
