# Supplementary material for: Elucidate microbial characteristics in a full-scale treatment plant for offshore oil produced wastewater
Source: PLoS One. 2021 Aug 12;16(8):e0255836. doi: 10.1371/journal.pone.0255836 (PMC8360554; doi:10.1371/journal.pone.0255836)
Supplement: Attachment — Submitted filename: Response to comments.docx [file pone.0255836.s006.docx]

Dear Editors and Reviewers:

Thank you for your letter and for the comments concerning our manuscript entitled “Elucidate microbial characteristics in a full-scale treatment plant for offshore oil produced wastewater” (PONE-D-21-08773). Those comments are all valuable and very helpful for revising and improving our paper, as well as the important guiding significance to our researches. We have studied comments carefully and have made correction which we hope meet with approval. Revised portion are marked in red in the paper.

The main corrections in the paper and the responds to the reviewer’s comments are as following:

**Reviewer 1:**

Recommendation: This manuscript elucidated the microbial composition and the potential microbial functions in a full-scale well-worked offshore oil produced wastewater treatment plant using 16S rDNA amplicon sequencing. In general, the finding of this work is interesting. However, the following comments should be addressed.

(1) The level of English should be improved and the data format should be unified throughout the manuscript.

Response:

We have polished our manuscript through the ShineWrite.com and have checked the words, sentences and data throughout the manuscript.

(2) In “Highlight” sections, their content should be rewritten and simplified for better description the manuscript.

Response:

Pg 2, Line 17. We have rewritten the “Highlight” according to the reviewer’s suggestion.

(3) If the abbreviation appears for the first time in the manuscript, please indicate its specific name. (like VFA, WWTS)

Response:

Pg 6, Line 121. “VFAs” appears for the first time and is indicated.

Pg 11, Line 216. WWTS appears for the first time and is indicated.

We checked other abbreviations and indicated their special names in the manuscripit.

(4) “NH3-N concentration in the water phase of ashore water-oil sample was 125.58 mg/L, after the anaerobic treatment the NH3-N concentration decreased to 56.72 mg/L”. Please explain how NH3-N was removed in ABR.

Response:

Based on the data of microbial community and functional annotation in the ABR (Figure 3 and 4), we could not find typical microbes and functions of ammonia oxidation. Therefore, we could not give a concrete microbial reason on the decrease of NH3-N concentration in the ABR. As we discussed in Line 176-186, we found the inconsistency and only could give a general reason of many relative factors that varied in biological wastewater treatment processes.

(5) Concentration of sulfate was increased in ABR, why? How COD was removed in ABR? Why was not methane produced in ABR?

Response:

Pg 17, from line 359-387, we discussed the sulfur cycle and typical microbes in the ABR, sulfur cycle involving sulfate reduction to sulfide (SO_4_^2−^→S^2−^) and subsequent sulfide oxidation to elemental sulfur (S^2−^→S_0_). Here, there wasn’t obvious microbial evidences for sulfate production (typical SOBs for sulfate). Uncultured Campylobacterales were dominant, however studies for their metabolism were few as the uncultured features. Oxygen introduction might be for chemical oxidation of sulfide，while the data we had was not adequate. Therefore, more studies are needed. Here we described/reported the microbial characteristics in the plant to provide a basic researching results for the following studies.

Pg 18, 380-387, we referred to COD removal in the ABR as sulfur cycle as the study by Yun et al., 2019.

Fig 4. We did not detected methane and Methanogen in the study, but we mentioned the potential metabolism of methane production in Fig 4. Studies on archaea is our next focus point.

(6) What was the physical processes?

Response:

Pg 101-113, Figure 1. We described and illustrated the each treatment process, before the wastewater coming to biotreatment, it flowed from the three separators, the horizontal oil eliminator, the vertical walnut shell filter and the settlement pond (SP). These are physical processes for oil produced wastewater.

(7) The salt concentration in the oil production wastewater is high. In the process of biological treatment of wastewater, how much influence does it have on the treatment efficiency of microorganisms?

Response:

Pg11, line 209-239. The salt concentration in the oil-produced wastewater is high as the seawater-flooding oil recovery and the produce of formation water. However, the influence of high salinity on microbes was low. As we found that a majority of the detected microbes in the plant were originated from oil reservoirs or marine-associated environments. Selective pressure imposed by environmental impactors shaped the microbial communities the plant.

(8) In three aerobic reactors, the diversity and functional similarities of microorganisms in three different aerobic treatments need to be discussed in more detail.

Response:

From Pg 20, Line 411. It is better to discuss the results in the three aerobic reactors in more detail. However, given the length of the manuscript, the numbers of dominant OTUs and the complex of microbial function net work, we summarized that different microbes play similar functions in the three aerobic parallel reactors in a whole. The discussion part showed the predominant microbial characteristics in the three APRS.

(9) The layout of Figure 3 May be improve and some signs overlap so the signs are not clear.

Response: The relative abundances of functions in some samples show close, some signs in the Figure 3 overlapped. we have used hollow/soil/dash sphere/ triangle and colors to identified different samples and have tried many combination schemes. The present layout might be the most optimal show for the data with a little overlap. We will provide a higher resolution image for publication.

(10) Please check “A majority of sequences detected by highthrough sequencing methods are identified with bacteria below to order lever that can not be affiliated with functional groups as many functions are only conserved at species or genus

level in FAPROTAX database. I”.

Response:

Pg 4 line 79-83. Despite FAPPOTAX apparent merits of constructing prokaryotic tax to metabolic or other ecologically relevant functions, it has limitations. The statement at line 84-88 was cited from the reference (Louca et al., 2016). We revised the sentence and added the reference.

Special thanks to you for your good comments

**Reviewer 2:**

Recommendation: In this study authors describe the biological treatment of oil-produced wastewater. To improve the treatment process and meet stringent effluent discharge limits, it is important to identify the microbial members found in these systems and understand their metabolic potential. The topic is interesting, the experiments are comprehensive, the findings are useful, for adding in-depth understanding to biological wastewater treatment. To make it better, I have some suggestions/questions as below:

(1) Pg 3, ‘…remains curtail to modern civilization..”

	Sentence unclear

Response:

Pg 3 Line 40, in the sentence, we revised “crutail” to “important”.

(2) Pg 5, In this work, we elucidated the structures and potential functions of bacteria in a full-scale offshore oil produced wastewater treatment plant based on highthrough 16S rDNA sequences.

	Do the authors mean 16S ribosomal RNA (rRNA) or recombinant DNA (rDNA)?

Response:

Pg 4, Line 72, here we mean16S ribosomal RNA (rRNA) gene.

(3) Pg 6, correct OIIME-1.6.0 Pipelines to QIIME-1.6.0…

Response:

Pg 7, Line 142, We have corrected “OIIME-1.6.0 Pipelines “ to “QIIME-1.6.0”.

(4) Pg 8, ‘NH3-N concentration in the water phase of ashore water-oil sample was 125.58 mg/L, after the anaerobic treatment the NH3-N concentration decreased to 56.72 mg/L, and after aerobic treatment the NH3-N concentration in the effluent below 1 mg/L (Table 1).’

	Ammonia usually has an inhibitory effect on anaerobic digestion but given that 334 mg/L COD reduction was observed, it might seem that the ammonia was either being stripped in gas phase or some microorganisms present in the digester could utilize ammonia since in section 3.4 there is mention of oxygen intrusion?

Response:

Pg 9 Line 176-178, we also found the the COD and NH3-N removal rates in our anaerobic and aerobic reactors were inconsistent with those in previous studies. However, based on the data of microbial community and functional annotation in the ABR (Figure 3 and 4), we could not find typical microbes and functions of anaerobic ammonia oxidation. Paradoxically, oxygen intrusion was mentioned as the appearance aerobic microbes. According to the data of detected microbes, we could not give a concrete microbial reason on the decrease of NH3-N concentration in the ABR and only could give a general reason of many relative factors that varied in biological wastewater treatment processes. More studied of uncultured microbes were needed.

(5) Pg 8, Section 3.2, It is important to statistically analyse changes in abundance of microbial community members (Fig 2) and metabolic functions (Fig 3) in different treatments and include it in discussions of sections 3.2 – 3.5.

Response:

We format the results and discussion in one section in our manuscript to describe the microbial characteristics of our studied samples. As the numbers of detected dominant microbes and functions were abundant, and considering the length limitation of the manuscript, we presented the results where they were used to each subtitle. Therefore, Fig 2 was used dispersedly at Pg 10, Lin2 203, Pg 14, Line 300-304, Pg 15 Line 335, Pg 20, 415-420, 427-429.

(6) Pg 9, ‘In addition, as seen in Fig. S1, only the rarefaction curve of AOW and SP tended to approach the saturation plateau. The rest of rarefaction curves show little sign of plateau, indicating that the biological treatment processes contained a somewhat broader phylogenetic diversity...’

	α-, β diversity measures will be more conclusive than rarefraction curves. Rarefraction curves not plateauing may also refer to insufficient depth of sequencing.

Response:

Pg 10, Line 211, It is true that rarefraction curves can show the diversity measures and the depth of sequencing. In the original version of our manuscript, we used the rarefraction curves to indicated that microbial diversity in biotreatment processes were higher than that in physical processes without considering the depth of sequencing. In order to avoid the dispute, we removed the results of rarefraction curves (Fig S1）.

(7) Pg 12, ‘…which could provide microbial data to the downstream treatment system, especially to those systems using biotreatments.’

	Sentence unclear

Response:

Pg 14, Line 285, removed the sentence.

(8) Pg 16, However, the aerobes and anaerobes co-existing ecology systems were reported popular in natural environments and could significantly enhance pollutants removal...

Pg 17, …as organic matters from wastewater are initially adsorbed to the activated sludge and are degraded by microbes inhabited in the inner of the sludge (Mario, et al., 2019).

	Above explanations can be improved by discussing factors that lead to ecological gradients in sludge and how this allows microorganisms to co-exist in these conditions.

Response:

Yes, it is true to make a good discussion point about relations between ecological gradients and microbial co-exist. However, our present version of the manuscript is very long, we have to soften some point.

(9) Pg 20 - Hyphomicrobiaceae-affiliated microbes inhabited in wastewater system were commonly detected to posse nitrogen removal enzymes and were closely related to nitrogen removal (Jia et al., 2019; Tomasek et al., 2017).

	Authors may also discuss that Hyphomicrobium-related species are also commonly found in denitrifying wastewater treatment systems as identified in:

a)	Lu, H., Chandran, K. and Stensel, D., 2014. Microbial ecology of denitrification in biological wastewater treatment. Water research, 64, pp.237-254.

Response:

Pg 223 Line 478-479, we added the reference.

(10) Pg 20 – “However, as the microbial communities of the studied reactors were so complex and consisted of a large percentage of uncultured candidate divisions, it was not easy to draw the potential metabolize network in each reactor. More studies associated strains isolation and function genes detection are therefore important to follow up.”

	Authors can mention that since many microorganisms could not be identified, it should be considered in future work on this topic. Further, strain isolation has several redundancies but new techniques such as long-read sequencing methods are being increasingly applied to identify microbial community members in wastewater treatment plants as shown in:

a)	Singleton, C.M., Petriglieri, F., Kristensen, J.M., Kirkegaard, R.H., Michaelsen, T.Y., Andersen, M.H., Kondrotaite, Z., Karst, S.M., Dueholm, M.S., Nielsen, P.H. and Albertsen, M., 2021. Connecting structure to function with the recovery of over 1000 high-quality metagenome-assembled genomes from activated sludge using long-read sequencing. Nature Communications, 12(1), pp.1-13.

b)	Arumugam, K., Bessarab, I., Haryono, M.A.S. et al. 2021. Recovery of complete genomes and non-chromosomal replicons from activated sludge enrichment microbial communities with long read metagenome sequencing. npj Biofilms Microbiomes 7, Nature Publishing Group, https://doi.org/10.1038/s41522-021-00196-6

Response:

Pg 24, Lin 492-494, following the comments, we revised the paragraph and added the references.

(11) Pg 20 – Conclusion: In this study, we have characterized the composition and interaction of the bacteria inhabiting in the full-scale…

>In my understanding, no interactions have been mentioned in the manuscript, rather the functional potential is discussed.

Response:

Pg 23, Lin 497, we revised the sentence as “In this study, we characterized the composition and potential functions of ....”

(11) Table 1. Correct to AWO

Response:

We checked all ashore oil-water (AOW)

(12) Fig 1. Can mention in footnote that 2 samples, ABRS and ABRW, were collected from ABR.

Response:

Fig 1. we revised “AWO” to “AOW” and mentioned ABRS (Sludge sample) and ABRW (Water sample)

(13) Fig 2. Different colours to be used for alphaproteobacteria and unidentified taxa.

Response: we used highcharts with dashes and colors

References – Please check the consistency of all references, including author names, journal names and titles.

Response: we checked all the references.

We tried our best to improve the manuscript and made some changes in the revised manuscript. These changes will not influence the content and framework of the paper.

We appreciate for Editors and Reviewers’ warm work earnestly, and hope that the correction will meet the approval.

Once again, thank you very much for your comments and suggestions.
